# Genome Integration and Excision by a New Streptomyces Bacteriophage, ϕJoe

**DOI:** 10.1128/AEM.02767-16

**Published:** 2017-02-15

**Authors:** Paul C. M. Fogg, Joshua A. Haley, W. Marshall Stark, Margaret C. M. Smith

**Affiliations:** a Biology Department, University of York, York, United Kingdom; b Institute of Molecular, Cell and Systems Biology, University of Glasgow, Glasgow, United Kingdom; McMaster University

**Keywords:** serine integrase, recombination directionality factor, integration vector, R4-like phage, Streptomyces venezuelae, Streptomyces coelicolor, mobile genetic elements, bacteriophage genetics

## Abstract

Bacteriophages are the source of many valuable tools for molecular biology and genetic manipulation. In Streptomyces, most DNA cloning vectors are based on serine integrase site-specific DNA recombination systems derived from phage. Because of their efficiency and simplicity, serine integrases are also used for diverse synthetic biology applications. Here, we present the genome of a new Streptomyces phage, ϕJoe, and investigate the conditions for integration and excision of the ϕJoe genome. ϕJoe belongs to the largest Streptomyces phage cluster (R4-like) and encodes a serine integrase. The *attB* site from Streptomyces venezuelae was used efficiently by an integrating plasmid, pCMF92, constructed using the ϕJoe *int-attP* locus. The *attB* site for ϕJoe integrase was occupied in several Streptomyces genomes, including that of S. coelicolor, by a mobile element that varies in gene content and size between host species. Serine integrases require a phage-encoded recombination directionality factor (RDF) to activate the excision reaction. The ϕJoe RDF was identified, and its function was confirmed *in vivo*. Both the integrase and RDF were active in *in vitro* recombination assays. The ϕJoe site-specific recombination system is likely to be an important addition to the synthetic biology and genome engineering toolbox.

**IMPORTANCE** Streptomyces spp. are prolific producers of secondary metabolites, including many clinically useful antibiotics. Bacteriophage-derived integrases are important tools for genetic engineering, as they enable integration of heterologous DNA into the Streptomyces chromosome with ease and high efficiency. Recently, researchers have been applying phage integrases for a variety of applications in synthetic biology, including rapid assembly of novel combinations of genes, biosensors, and biocomputing. An important requirement for optimal experimental design and predictability when using integrases, however, is the need for multiple enzymes with different specificities for their integration sites. In order to provide a broad platform of integrases, we identified and validated the integrase from a newly isolated Streptomyces phage, ϕJoe. ϕJoe integrase is active *in vitro* and *in vivo*. The specific recognition site for integration is present in a wide range of different actinobacteria, including Streptomyces venezuelae, an emerging model bacterium in Streptomyces research.

## INTRODUCTION

Over the past few decades, serine integrases have become widely established as tools for genome engineering and synthetic biology (1, 2). Serine integrases are phage-encoded DNA site-specific recombinases that mediate recombination between two short (<50 bp) sequences. The integration reaction occurs during the establishment of lysogeny, during which the integrase causes a single crossover between the *attB* site on the bacterial chromosome and the *attP* site on the circularized phage genome, leading to the integrated phage DNA flanked by the recombinant sites *attL* and *attR* (1, 3). Integrase dimers bind to the two *att* sites and produce double-strand breaks with 2-bp overhangs (3, 4); the cut ends are then exchanged, and the DNA backbone is religated to produce the recombinant products (5). The *attL* and *attR* sites each contain reciprocal halves of the *attP* and *attB* sites (6). As integrases are unable to use *attL* and *attR* as substrates without an accessory protein, a recombination directionality factor (RDF), the integrated phage genome is stable until the RDF-encoding gene is expressed during prophage induction (3). Recombination between *attL* and *attR* is the excision reaction and is essentially the reverse of integration, releasing the phage genome and reforming *attP* and *attB*. While only integrase is required to mediate integration, excision requires both integrase and the RDF. Genome engineers have exploited these systems to integrate genes of interest into a specific site on the chromosome, which can either be the endogenous *attB* or an introduced *attB* or *attP* used as a docking site (1). The simplicity of the serine integrase-mediated site-specific recombination systems means that they are reliably portable to heterologous hosts where DNA can be integrated stably and in single copy.

The simple requirements for serine integrases make them amenable to a wide variety of applications. The earliest examples of this were to integrate an *attP* plasmid into a target genome containing the cognate *attB* (or *vice versa*) (7), allowing stable delivery of genes into diverse species, including bacteria (6, 8–10), mice (11), mosquitoes (12), and humans (13). More complex genetic engineering approaches use integrases in *in vitro*-ordered assembly of multiple DNA fragments (14, 15). *In vivo* genome manipulations can also be achieved either by iterative rounds of recombination (16, 17) or multiplexing orthogonal integrase/*att* sites (18). Integrase-mediated DNA rearrangements can also be used to provide permanent genetic memory in novel types of biosensors (19, 20). Some applications, such as *post factum* modifications (15) or biocomputing (19, 21), need controlled excision, and this requires integrase and its cognate RDF. The RDF binds directly to the integrase protein and is thought to induce a conformational change that allows *attL* and *attR* to be used as recombination substrates while inhibiting recombination of *attB* and *attP* (22, 23).

A limiting factor for the use of serine integrases for complex multiplexed applications is the number of well-characterized integrases and, perhaps more pressingly, RDFs. Only seven integrase-RDF pairs have been characterized to date (from phages TP901-1 [24], ϕC31 [22], ϕBT1 [25], Bxb1 [23], ϕRv1 [26], and SPBc [27], and from the excisive element of Anabaena and Nostoc cyanobacterial species [28]), but many more integrases have been studied without their RDFs (1, 2, 29–31). Integrase genes are easily identified by comparative sequence analysis, and when the integrase is prophage encoded, the attachment sites can also be predicted (31). RDFs, however, are far more difficult to predict, because known examples share little sequence homology, vary markedly in size, and differ in gene location in phage genomes (1). Expansion of the available arsenal of serine integrases and RDFs is desirable to enable advanced synthetic biology applications.

Phages that encode serine integrases are prevalent in Gram-positive bacteria, and in particular in actinobacteria. Here, we describe a newly isolated Streptomyces phage, ϕJoe, and its serine integrase (Int) that is only distantly related to characterized integrases. ϕJoe Int is active *in vivo* in Streptomyces and E. coli, and the integrase protein is readily purified and is able to carry out efficient *in vitro* recombination. We also describe the ϕJoe RDF, a 6.8-kDa protein that is able to promote excisive recombination and inhibit integration.

## RESULTS AND DISCUSSION

### Isolation of actinophage ϕJoe and genome sequence.

**FIG 1:**
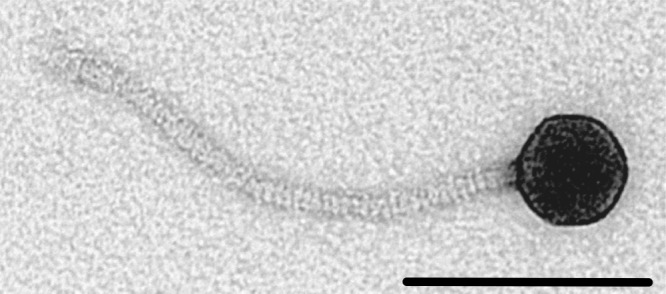
A ϕJoe virion imaged by transmission electron microscopy. Viral particles were negatively stained with uranyl acetate, and this image was taken at ×220,000 magnification. The scale bar represents 100 nm.

Raw soil samples were enriched for environmental phage using S. coelicolor strain M145 as a propagation host. The phage chosen for further analysis, ϕJoe, is a siphovirus with a capsid diameter of 46.5 nm (standard deviation [SD], 1.6 nm; *n* = 9) and a long flexible tail of 199.5 nm (SD, 12 nm; *n* = 8), with clear striations visible in most images (Fig. 1). ϕJoe is able to plaque on a broad range of Streptomyces hosts, producing lytic infection of seven out of nine species tested (Table 1). Saccharopolyspora erythraea (formerly Streptomyces erythraeus) and Streptomyces venezuelae were resistant to infection.

**TABLE 1:**
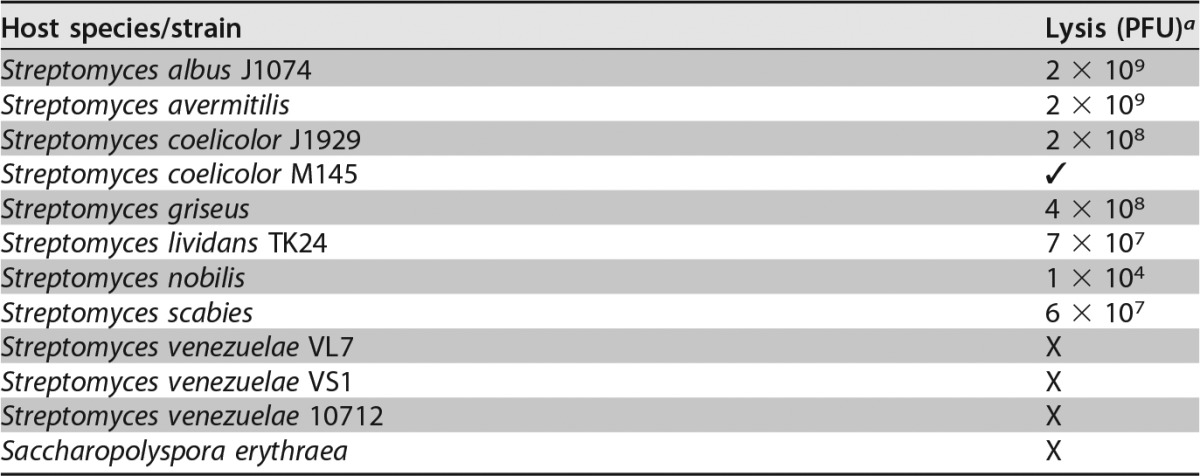
ϕJoe host range

a PFU per milliliter values quoted are illustrative of the relative plaquing efficiencies when challenged with the same phage stock propagated on S. coelicolor J1929. ✓, the phage could produce plaques on this strain, but the PFU/ml value was not calculated; X, we were not able to produce plaques for the indicated strain.

**FIG 2:**
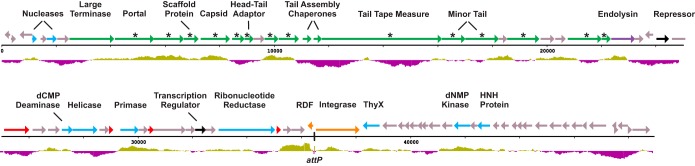
Schematic of the ϕJoe genome. The genome is 48,941 bp in length. ORFs were predicted using GeneMark and Glimmer and then manually curated. ORFs are labeled and color-coded based on their predicted function. Orange, recombination; cyan, metabolism and DNA processing/replication; green, structural proteins; purple, lysis; black, regulatory; gray, hypothetical proteins with no known function; red, candidate RDF genes. Genes marked with an asterisk encode structural proteins that were detected by MS/MS. The histogram below the genome contains purple bars to indicate below-average G+C content (65.5%) and green bars to indicate above-average G+C content (1,000-nt window size, 20-nt step).

**FIG 3:**
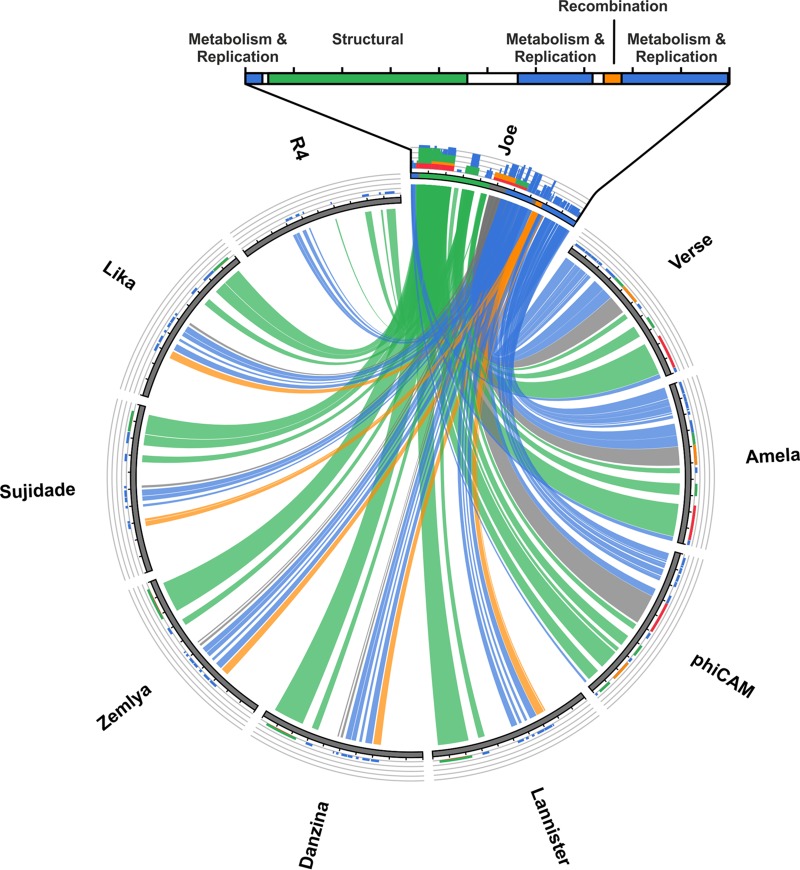
Circos plot of the ϕJoe genome versus nine related phages. A BLASTN comparison was carried out for ϕJoe, the five sequenced phages with a ϕJoe-like integrase, the three closest whole-genome matches, and the well-characterized R4 phage. The E value cutoff was set to 1 × 10^−100^ and the high-scoring segment pairs (HSPs) to 100; ribbons are colored by genomic regions as defined in Fig. 1 and depicted above the Circos plot. The histograms above each genome are colored to reflect relative homology to the ϕJoe sequence based on BLAST score (red > orange > green > blue).

Genomic DNA was extracted from high-titer ϕJoe suspensions (>10^10^ PFU/ml) and sequenced on the Illumina MiSeq platform with 2,542× coverage. The phage genome is 48,941 bases (accession no. KX815338), with a G+C content slightly lower than that of the host bacteria, at 65.5% compared to ∼72% for most Streptomyces species. BLASTn was used to measure nucleotide identity for the closest relatives to ϕJoe; the generalized transducing phage ϕCAM (32) and two newly sequenced Streptomyces phages, Amela and Verse (Fig. S2), are 73, 76, and 76% identical, respectively, in global alignments. The ϕJoe genome contains 81 predicted open reading frames (Fig. 2), the majority of which have amino acid sequences similar to those of the three phages mentioned above and the well-characterized R4 phage (33). Notably, similarity to ϕJoe integrase (gp53) is absent from each of the closest genome matches but is instead present in several more distantly related phages (Fig. 3), indicative of phage mosaicism (34). Specifically, ϕJoe integrase is homologous to the uncharacterized integrases from five complete phages: Lannister (78% amino acid identity), Zemyla (74%), Danzina (73%), Lika (73%), and Sujidade (73%) (Fig. 3). Comparison to known integrases suggests that the catalytic serine is likely to be at position 46 in the protein sequence (VRL**S**VFT).

Purified phage particles were submitted for shotgun liquid chromatography-tandem mass spectrometry (LC-MS/MS) analysis to determine the structural proteome. At least one peptide match was detected from 14 ϕJoe gene products, five of which have predicted functions: portal, capsid, tail tape measure, scaffold, and head-tail adaptor (Fig. 2 and Table S1). The remaining nine gene products have no known function, but all cluster close to the predicted structural genes within a region of the genome spanning ∼21 kbp.

### Characterization of ϕJoe integrase and attachment sites.

**FIG 4:**
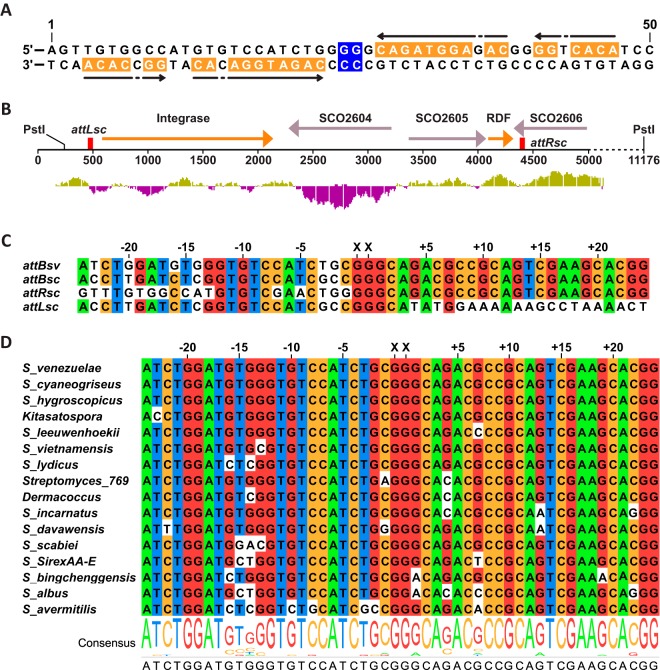
ϕJoe attachment sites and integration sites. (A) Diagram of ϕJoe *attP* showing the central dinucleotides (blue) and imperfect inverted repeats (orange and arrows). (B) Schematic of the genomic context of the two S. coelicolor integration sites (*attLsc* and *attRsc*, red boxes) used by the ϕJoe-integrating plasmid pCMF92. The location of the PstI sites used for identification of the *att* sites are shown. The DNA between the *attLsc* and *attRsc* sites is an apparent mobile genetic element, with homologous integrase and RDF genes (orange arrows) to those of ϕJoe. (C) Alignment of S. venezuelae *attB* (*attBsv*) with the two S. coelicolor att sites (*attRsc* and *attLsc*) and the reconstituted *attB* site (*attBsc*) that would be produced by excision of the DNA between *attRsc* and *attLsc*. (D) Alignment of closely related *attB* sites identified by a BLASTN search against the nonredundant GenBank database. Hits were first filtered for matches of at least 80% and then for an E value of <1 × 10^10^ and a bit score of >75. (C and D) Nucleotide positions are shown as distance from the crossover dinucleotides (XX).

For most phage-encoded integration systems, the *attP* site lies adjacent to the *int* gene encoding the integrase. The *attP* sites for serine integrases are characteristically about 45 to 50 bp in length and contain inverted repeat sequences flanking a spacer of approximately 20 bp (3, 35). Examination of the ϕJoe genome identified a candidate *attP* site located 18 bp upstream of the *int* gene. A plasmid, pCMF92, was constructed by replacing the ϕC31 *int-attP* locus from the widely used integrating vector pSET152 with the ϕJoe *int-attP* locus (Fig. S1). Integration of pCMF92 would confirm whether the integrase is functional and the nature of the *attP* site, and, by rescuing the DNA flanking the integrated plasmid, the identity of the *attB* site could be deduced (Fig. 4). pCMF92 was introduced into S. coelicolor J1929 and S. lividans TK24 by conjugation, and apramycin-resistant colonies were obtained, but the frequencies were low (10^−4^ to 10^−5^ exconjugants/CFU) compared to other integrating vectors (10^−2^ to 10^−3^ exconjugants/CFU) (9, 18). To test whether integration was site specific, four S. coelicolor::pCMF92 cell lines were amplified from independent exconjugants, and the genomic DNAs were analyzed by Southern blotting using a probe derived from the ϕJoe *int* gene. In the four cell lines, pCMF92 had integrated into one of two different integration sites, as revealed by hybridization of the probe to two different restriction fragments (data not shown).

We then sought to characterize the two integration sites for pCMF92 in S. coelicolor by rescuing the integrated plasmids along with flanking DNA into E. coli. In pCMF92, there is 3.9 kbp of DNA between the ϕJoe *attP* site and the PstI cleavage site that contains the plasmid origin of replication and the apramycin resistance gene (Fig. S1). Genomic DNA from two S. coelicolor::pCMF92 cell lines, each containing pCMF92 integrated into one of the two different integration sites, was digested with PstI endonuclease, self-ligated, and introduced into E. coli DH5α by transformation. The rescued plasmids were sequenced over the recombination sites to validate the nature of the ϕJoe *attP* site and to identify the chromosomal positions of the two S. coelicolor integration sites. The ϕJoe *attP* site was confirmed to be ≤50 bp, and the 5′-GG dinucleotide at the center of an imperfect inverted repeat is predicted to be where the crossover occurs (Fig. 4A).

**FIG 5:**
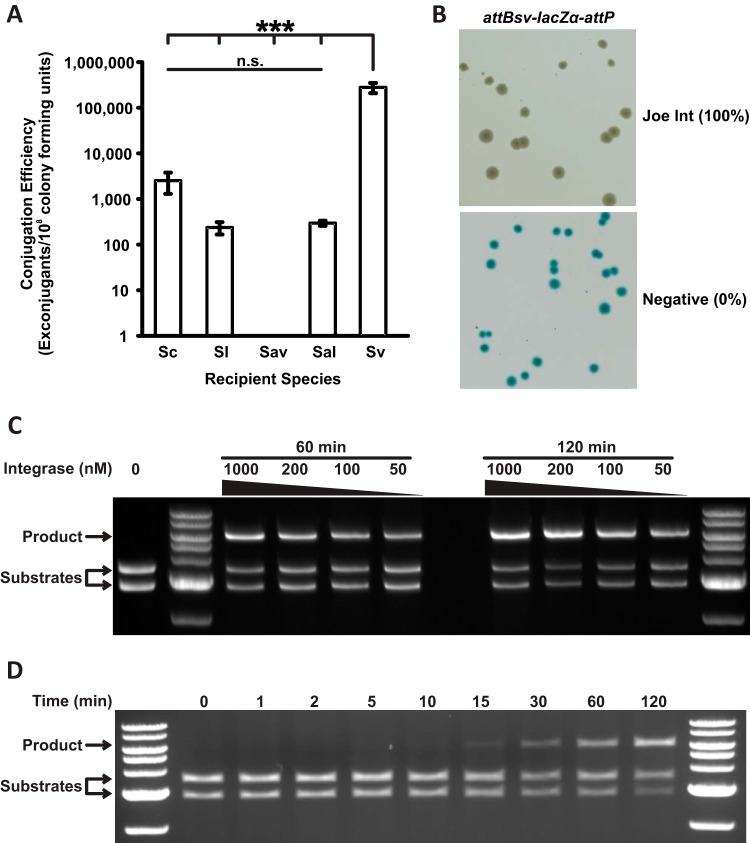
Activity of ϕJoe integrase *in vivo* and *in vitro*. (A) Conjugation efficiency of an integrating vector, containing ϕJoe *int* and *attP*, into five recipient species: Streptomyces coelicolor (Sc), S. lividans (Sl), S. venezuelae (Sv), S. albus (Sal), and S. avermitilis (Sav). Levels of significance for S. venezuelae versus all other species in a one-way analysis of variance (ANOVA) were a *P* value of <0.001 (3 asterisks); all other comparisons were nonsignificant (n.s.). Error bars are standard deviation (Sc, *n* = 5; Sv and Sl, *n* = 3; Sal and Sav, *n* = 2). (B) Representative image of an *in vivo* integration assay to assess *attBsv-attP* recombination by ϕJoe integrase (pCMF107) and a negative control (pBAD-HisA). Recombination leads to deletion of an intervening *lacZ*α gene and white colonies, and inactivity produces blue colonies. Integration efficiency is shown in parentheses (*n* = 3). (C) Representative image of *in vitro* recombination of two substrate plasmids, *attP* (pCMF91) and *attBsv* (pCMF95), to produce the cointegrant plasmid pCMF98. The concentration of ϕJoe integrase and incubation time for each reaction are indicated above the gel. (D) Time course for the integration reaction shown in panel C.

The two S. coelicolor integration sites for pCMF92 are located 3.9 kbp apart, separated by an apparent mobile genetic element comprising *sco2603*, encoding a putative serine integrase with 68% identity to ϕJoe integrase, and two further genes (Fig. 4B). Its product, SCO2603, is 68% identical to ϕJoe integrase. We hypothesized that the ϕJoe-integrating plasmid is inefficient in S. coelicolor because an ancestral and optimal *attB* site is occupied by the SCO2603-encoding element. The two integration sites for pCMF92 in S. coelicolor were therefore called *attLsc* and *attRsc* to reflect the provenance of the sites containing the mobile element. To test this hypothesis, the sequence of the ancestral *attB* site, *attBsc*, was predicted by removing the sequence between *attLsc* and *attRs*c, including the *attP* moieties that would have originated from the inserted mobile element (Fig. 4C). The reconstituted *attBsc* was used to interrogate the GenBank Streptomyces database for closely related extant sequences. Three species were chosen from the top 10 hits returned (S. avermitilis, S. albus, and S. venezuelae; Fig. 4D) and assayed for *in vivo* integration efficiency. S. venezuelae was the only host to support highly efficient integration after conjugation with pCMF92, at 160-fold greater frequency than S. coelicolor and 1,600-fold greater frequency than S. lividans (Fig. 5A). The integration frequencies for pCMF92 into S. venezuelae are similar to those reported for other characterized serine integrases (9, 18), and we demonstrate below that the *attB* site from S. venezuelae, *attBsv*, is indeed used efficiently by ϕJoe integrase. Plasmid pCMF92 could therefore be used as a new integrating vector for use in this newly emerging model system for Streptomyces research.

The S. venezuelae *attBsv* site was used as a BLASTn query to estimate the prevalence of potential ϕJoe insertion sites in sequenced species. In many instances, each half of the query sequence matched separate locations in the target genome, suggesting that ϕJoe-like *attB* sites are frequently occupied by either a prophage or a mobile element similar to that observed in S. coelicolor J1929. Hits were subsequently filtered for matches of at least 80% coverage, with an E value of <1 × 10^−10^ and a bit score of >75, which revealed numerous apparently unoccupied ϕJoe *attB* sites in diverse Streptomyces, Kitasatospora, and Dermacoccus species (Fig. 4D). Generally, the *attB* site for ϕJoe and the SCO2603 integrase-encoding elements are located 74 bp from the end of an open reading frame (ORF) encoding an SCO2606-like predicted B12 binding domain-containing radical *S*-adenosylmethionine (SAM) protein. Insertions this close to the end of an ORF may not necessarily cause loss of function of the gene product, and this could explain the prolific number of mobile elements that use this locus as an insertion site. Other than the recombination genes, the genetic content of the mobile elements located here varies markedly in different bacterial species (Fig. S2). Some Streptomyces strains have an SCO2603-containing genetic element almost identical to that of S. coelicolor J1929 (e.g., WM6391), others have no genes other than the recombination genes (e.g., NRRLF-5123), and some contain up to 40 kbp between the predicted *attL* and *attR* sites (Fig. S2).

### ϕJoe integrase catalyzes efficient *in vivo* and *in vitro* integration.

In order for an integrase to have broad appeal as a bioengineering tool, it must be functional in heterologous hosts. As a proof of principle, we tested the activity of ϕJoe integrase in E. coli by cloning the integrase gene into an arabinose-inducible expression vector, pBAD-HisA, to produce pCMF107. Meanwhile, we constructed a reporter plasmid, pCMF116, containing the E. coli *lacZ*α gene flanked by ϕJoe *attBsv* and *attP* sites in head to tail orientation (Fig. S3). Both plasmids were introduced into E. coli TOP10 cells (Invitrogen) by cotransformation and plated on selective agar plates containing 0.2% l-arabinose and 80 μg/ml 5-bromo-4-chloro-3-indolyl-β-d-galactopyranoside (X-Gal). pBAD-HisA lacking an insert was used as a negative control. All of the transformants were white in the presence of ϕJoe *int*, indicating efficient recombination between the *attBsv* and *attP* sites, leading to loss of the *lacZ*α gene (Fig. 5B and S3). ϕJoe integrase and its cognate *attBsv* and *attP* sites are, therefore, active in E. coli.

Another key application for serine integrases is for *in vitro* combinatorial assembly of genes for optimizing the expression of metabolic pathways (14, 15). In this application, different integrases are used to join (by recombination) specific pairs of DNA fragments tagged with their cognate attachment sites. In theory, this procedure can be multiplexed to assemble many DNA fragments together using different orthogonally acting integrases. The aim is to generate artificial operons with defined or random order. To test the suitability of ϕJoe Int for *in vitro* recombination reactions, the integrase gene was cloned into the His tag expression vector pEHISTEV and purified after overexpression in E. coli. *In vitro* recombination assays were carried out with ϕJoe *attP* (pCMF91) versus each of *attBsc*, *attLsc*, *attRsc*, and *attBsv* (pCMF97, pCMF90, pCMF94, and pCMF95, respectively) and using a range of ϕJoe integrase concentrations. Successful recombination between attachment sites produces a cointegrant plasmid, which can be distinguished from the substrate plasmids by restriction digestion and agarose gel electrophoresis (Fig. S3). In this assay, recombination was undetectable when *attLsc* (pCMF90) or *attRsc* (pCMF94) was used with *attP* (pCMF91) as the substrate. A small amount of recombination was observed (≤2%, Fig. S4) when the reconstituted *attBsc* (pCMF97) was used with *attP* (pCMF91). However, consistent with the observations in E. coli and in Streptomyces, the S. venezuelae *attBsv* site (pCMF95) was a highly efficient substrate for recombination with the ϕJoe *attP* site. ϕJoe integrase was effective over a broad range of concentrations (50 to 1,000 nM) (Fig. 5C and S4). Using 200 nM integrase, detectable recombination product was produced after ∼10 to 15 min, and after 2 h, approximately 70% of the substrate molecules were converted to product (Fig. 5C and D).

There are only 6 bp that differ between *attBsc* and *attBsv*, and all the differences are on the left-hand arm of the *attB* sites (Fig. 4C). Previously, a mutational analysis of the ϕC31 *attB* site showed that mutationally sensitive bases occur 2, 15, and 16 bases to either side of the crossover dinucleotide (36). As two of the differences between *attBsc* and *attBsv* are also 2 and 16 bases from the putative crossover 5′-GG (Fig. 4C), these base pair differences might account for the poor activity of *attBsc* in the *in vitro* assays.

### Identification and validation of the ϕJoe RDF protein, gp52.

Although there are dozens of serine integrases that have been described in the literature, there are only seven published RDFs for serine integrases (ϕC31 gp3 [22], ϕBT1 gp3 [25], Bxb1 gp47 [23], TP901 ORF7/Xis [24], Anabaena/Nostoc XisI [28], SPBc SprB [27], and ϕRv1 Rv1584c/Xis [26]). The Bxb1 and ϕC31 RDFs are among the largest of these RDF proteins (approximately 27.5 kDa and 250 amino acids), and their genes are located in proximity to the phage DNA replication genes. Both RDFs have functions during phage replication in addition to acting as RDFs, but they are evolutionarily unrelated (25, 37). The RDFs from ϕBT1 and another ϕC31-like phage, TG1, are close relatives of the ϕC31 RDF at the sequence level (85% and 59% identical, respectively); furthermore, the ϕBT1-encoded RDF acts on ϕC31 integrase and *vice versa* (25). The ϕRv1 and SPBc RDFs are located within 1 or 2 ORFs of the *int* gene, a feature which is reminiscent of the *xis* genes that act with tyrosine integrases. ϕRv1, SPBc, TP901, and Anabaena/Nostoc RDFs are much smaller proteins than ϕC31 gp3 or Bxb1 gp47 (58 and 110 amino acids). Given the variation in RDF size, sequence, and genomic location, there are no sound generalizations yet for identifying new RDFs in phage genomes.

**FIG 6:**
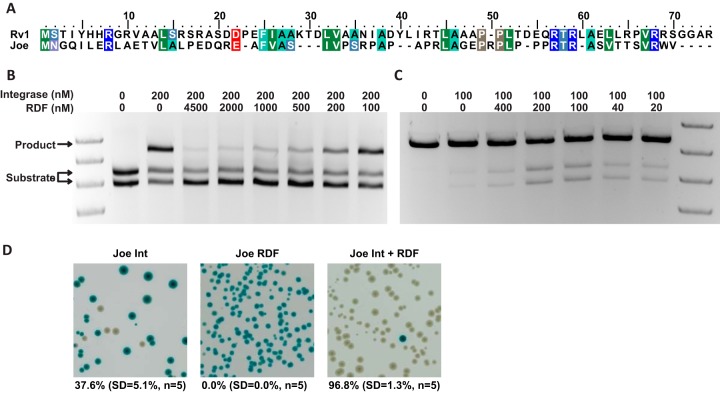
Identification of the ϕJoe RDF, gp52. (A) Alignment of ϕJoe and RV1 RDFs, colored using the BLOSUM62 scheme. (B) Representative agarose gel showing *in vitro* inhibition of integration by ϕJoe RDF. The concentrations of ϕJoe integrase and RDF for each reaction are indicated above the image. Reactions were stopped after 2 h and linearized using XhoI. (C) Representative agarose gel showing *in vitro* excision reactions catalyzed by ϕJoe integrase and RDF. The concentrations of ϕJoe integrase and RDF for each reaction are indicated above the image. Reactions were stopped after 2 h and linearized using XhoI. (D) *In vivo* excision assay to assess *attLsv* × *attRsv* recombination by ϕJoe integrase alone, ϕJoe RDF alone, and ϕJoe integrase coexpressed with the RDF. Recombination leads to deletion of an intervening *lacZ*α gene and white colonies, and inactivity produces blue colonies. Expression from the T7 promoter successfully achieved almost complete excision activity for ϕJoe Int + RDF.

A list of four candidate genes (*g40*, *g43*, *g49*, and *g52*) for the ϕJoe RDF was drawn up based on comparable size to known small RDFs and genomic location (i.e., not located among the late/structural genes) (Fig. 2). One of the potential RDF genes (*g52*) is adjacent to *int* in the ϕJoe genome, but it is transcribed divergently, with the *attP* site situated between *int* and *g52* (Fig. 2). Unlike the other candidate RDFs, gp52 homologues are only found in those phages with ϕJoe-like integrases (Fig. 3), and phylogenetic analysis of gp52 and the integrase indicated that the two proteins have followed a parallel evolutionary path (Fig. S5). Pairwise alignment of the 6.8-kDa (62 amino acids) gp52 protein with other known small RDFs revealed homology with ϕRv1 RDF (25.7% identity and 35.1% similarity; Fig. 6A). Also, examination of the mobile elements that have inserted into the *attB* sites in S. coelicolor and other Streptomyces spp. revealed that they also contain a gene encoding a gp52 homologue in a similar genetic context, i.e., the *int* and *g52* genes are adjacent to the *attL* and *attR* sites, respectively, and would flank *attP* after excision (Fig. 4B and S2). The predicted secondary structure of ϕJoe gp52 contains an alpha-helix in the N-terminal region, a beta-sheet in the C-terminal region, and an unstructured region between (Fig. S6). Alignment of the ϕJoe-like RDFs found in intact phages and the RDFs found in the SCO2603-encoding mobile elements indicated that both of the structured regions are well conserved, particularly the putative alpha-helix, but the center of the protein is variable (Fig. S6).

RDFs are able to influence integrase-catalyzed recombination in two ways; they activate the *attL* × *attR* reaction to regenerate *attP* and *attB* (excision), and they inhibit the *attB* × *attP* integration reaction (22, 23). We were unable to produce sufficient soluble gp52 protein for *in vitro* assays when expressed with a simple histidine tag; however, a maltose-binding protein (MBP)-gp52 fusion protein was more soluble. We tested the ability of MBP-gp52 to inhibit integration by titrating the protein against a fixed concentration of integrase at MBP-gp52/Int ratios of 1:2 to 22.5:1. When the MBP-gp52 was in excess, integration was repressed to less than 10%; however, at less-than-equimolar concentrations, recombination was equivalent to the control in which no MBP-gp52 was added (Fig. 6B). These results are similar to observations for ϕC31 and Bxb1 integrases and their cognate RDFs, gp3 and gp47 (22, 23).

To test the ability of gp52 to activate an excision reaction, a plasmid containing the cognate *attLsv* and *attRsv* sites was produced, pCMF98 (Fig. S3). The MBP-gp52 protein was unable to promote efficient excision under any conditions tested (not shown). Removal of the MBP tag using 3c protease increased excision activity, but the reaction was still inefficient after 2 h of incubation (Fig. 6C). Longer incubations of 5 to 20 h further increased the amount of substrates converted to product up to 45%, but it also led to significant amounts of excision products (10 to 20%) by the integrase alone. Thus, in comparison to the activity of other RDFs, gp52 has rather poor activity; ϕC31 gp3 activates approximately 60 to 80% conversion of the *attL* × *attR* substrates to products (22), and similar results are obtained with other RDFs (23, 25, 26).

To test the excision ability of ϕJoe gp52 *in vivo*, a *g52-int* coexpression operon was designed in which *int* and *g52* were located directly downstream of the T7 promoter and ribosome binding site (RBS) in the expression vector pEHISTEV to produce pCMF117. A reporter plasmid, pCMF103, was produced containing the *lacZ*α gene flanked by ϕJoe *attLsv* and *attRsv* sites (Fig. S3). pCMF117 and pCMF103 were introduced into E. coli BL21(DE3) cells by cotransformation and plated onto LB agar supplemented with 0.5 mM isopropyl-β-d-thiogalactopyranoside (IPTG) to induce expression of the *g52-int* operon (30). The reporter plasmid was then extracted from the BL21(DE3) transformants and introduced into E. coli DH5α to determine the percentage of plasmids that had undergone *attLsv* × *attRsv* recombination and had lost the *lacZ*α gene. As controls, plasmids expressing either only integrase (pCMF87) or only gp52 (pCMF100) were also introduced together with the reporter (pCMF103) into BL21(DE3), and the assay was repeated using the same procedure. When ϕJoe integrase alone was expressed, excision occurred at a frequency of 37.6% (SD, 5.1%; *n* = 5), but when coexpressed with gp52, the frequency rose to 96.8% (SD, 1.3%; *n* = 5) (Fig. 6D). Expression of gp52 without integrase led to no detectable excision events (Fig. 6D). Although overall recombination *in vivo* was higher than that *in vitro*, the relative levels of *attLsv* × *attRsv* recombination by ϕJoe integrase alone and ϕJoe integrase with gp52 were comparable. Taken together, the *in vivo* and *in vitro* data indicate that ϕJoe gp52 has RDF activity.

The observation that ϕJoe integrase has a basal level of excision activity in the absence of its RDF is highly unusual for a phage-encoded integrase, and further study may provide novel insights into the mechanism and evolution of the serine integrases. Streptomyces phage ϕBT1 integrase was shown to catalyze bidirectional recombination, albeit at extremely low levels (38). The archetypal ϕC31 integrase is only able to mediate *attL* × *attR* recombination in the absence of gp3 when certain mutations are introduced just upstream or within a motif, the coiled-coil motif, required for subunit-subunit interactions during synapsis of DNA substrates (39). The coiled-coil motifs are also thought to play a role in inhibiting recombination between *attL* and *attR* in the absence of the RDF; the ϕC31 IntE449K mutation or its RDF, gp3, relieves this inhibition (35, 39–41). Three independent structural predictions indicate the presence of a coiled-coil domain in the ϕJoe Int C-terminal domain (A395-T453, Fig. S7). The high basal excision activity of ϕJoe integrase could be due to incomplete inhibition of synapsis by the coiled-coil motif when integrase is bound to *attL* and *attR*, reminiscent of the hyperactive ϕC31 mutant IntE449K (39). Natural bidirectional large serine recombinases include the transposases TnpX (42) and TndX (43) from clostridial integrated conjugative elements (ICEs); ϕJoe integrase could be an evolutionary intermediate between these bidirectional recombinases and the highly directional recombinases, such as ϕC31 and Bxb1 integrases. Our data show that, under the *in vitro* conditions used, gp52 was highly effective at inhibiting integration by ϕJoe integrase but only weakly activated excision. It remains to be seen whether this system, with its unusual properties, is sufficiently robust to regulate phage genome integration and excision according to the developmental choices of ϕJoe.

The properties of the ϕJoe integrase and gp52 are compatible with some of the existing applications for serine integrases, but they could also present opportunities for new applications. ϕJoe integrase is highly efficient in integration assays *in vivo* and *in vitro*, and in *in vivo* excision when the RDF is present. In *attB* × *attP* integration assays, the yield of products by ϕJoe integrase was comparable to that of well-established integrases, such as that of ϕC31 or Bxb1. Furthermore, ϕJoe integrase is active in buffers compatible with other characterized integrases, indicating that it could be used in DNA assembly procedures in combination with other integrases. Although yet to be tested, assemblies generated with ϕJoe integrase could later be used as substrates for modification by ϕJoe integrase in a single step. The innate excision activity of ϕJoe integrase could excise a fragment flanked by *attLsv* or *attRsv* sites and, in the same reaction, replace it via an integration reaction. ϕJoe integrase could therefore provide a more streamlined tool than the existing requirement for two steps by the more directional integrases, such as those from ϕC31 and Bxb1 (15). Furthermore, given that ϕJoe Int can mediate basal levels of excision in the absence of RDF, integrating plasmids based on ϕJoe *int-attP* may display a degree of instability. Selection for the plasmid marker would ensure plasmid maintenance when desired, but if the plasmid is easily lost without selection, this trait could be desirable if there is a need to cure the strain of the plasmid or during studies on synthetic lethality.

### Conclusions.

On the basis of sequence and genome organization, phage ϕJoe is a member of a large cluster of R4-like Streptomyces phages. Its closest relatives at the nucleotide level are Streptomyces phages Amela and Verse, with very high levels of nucleotide identity in the regions containing essential early and structural genes. However, ϕJoe integrase is more closely related to the integrases from five other R4-like cluster phages: Lannister, Danzina, Zemlya, Lika, and Sujidade. At this time, the majority of Streptomyces phages belong to the R4-like cluster phages, but there is a continuum of relatedness throughout the cluster; for example, R4 is a more distant relative to ϕJoe than any of the other phages mentioned above.

We identified the RDF for ϕJoe integrase on the basis of its gene location, small size, and distant similarity to another known RDF, Rv1584c. Although this identification was relatively straightforward, it is not clear yet how general such an approach might be. The activities of ϕJoe integrase and RDF contribute to the growing number of complete serine integrase site-specific recombination systems that are available for use in synthetic biology applications. The ϕJoe *int-attP* plasmid, pCMF92, also adds to the number of useful integrating vectors for use in Streptomyces species. However, and unusually for a phage integrase, ϕJoe Int displays a significant level of excisive recombination in the absence of its RDF while still being efficient at mediating integration. This bidirectional property could be applied in new ways in future applications of serine integrases.

## MATERIALS AND METHODS

### Growth media.

Escherichia coli strains were generally grown in LB, except where otherwise noted. Antibiotics were added for selection where appropriate (apramycin, 50 μg/ml; chloramphenicol, 50 μg/ml; kanamycin, 50 μg/ml; ampicillin, 100 μg/ml). Preparation of competent cells and transformation of E. coli were performed as described by Sambrook et al. (44). Streptomyces strains were grown on mannitol soya agar (45) supplemented with 10 mM MgCl_2_, for plating conjugation mixtures, and antibiotics, where required (apramycin, 50 μg/ml; nalidixic acid, 25 μg/ml).

### Phage isolation.

The procedures for isolation, plating, and titration of phage with Streptomyces as the isolation host are described in detail by Kieser et al. (45). Raw soil samples were enriched for environmental phage using S. coelicolor M145 as a propagation host (46). Briefly, 3 g of soil was added to 9 ml of Difco nutrient (DN) broth (BD Diagnostics, Oxford, UK) supplemented with 10 mM CaCl_2_, 10 mM MgSO_4_, and 0.5% glucose. Streptomyces spores were added to a concentration of 10^6^ CFU/ml and incubated at 30°C with agitation for 16 h. Soil and bacteria were removed by centrifugation and filtration through a 0.45-μm-pore filter. A dilution series of the filtrate in SM buffer (100 mM NaCl, 8.5 mM MgSO_4_, 50 mM Tris-HCl [pH 7.5], 0.01% gelatin) was plated with S. coelicolor spores to isolate single plaques. Phage were recovered from single well-isolated plaques by single-plaque soak-outs in DN broth and replated with the host strain for three rounds of plaque purification. A high-titer phage preparation was generated from plates inoculated with sufficient PFU to generate almost-confluent lysis (45). The phage suspensions were filtered, pelleted by ultracentrifugation, and resuspended in 0.5 ml of SM buffer (47). The concentrated phages were further purified by cesium chloride isopycnic density gradient centrifugation (48).

### Next-generation sequencing.

Phage DNA was extracted by phenol-chloroform purification (44), and the presence of pure phage DNA was confirmed by restriction digestion. Phage DNA was sequenced and assembled in collaboration with Darren Smith at NU-OMICS (Northumbria University, Newcastle, UK). DNA was prepared for next-generation sequencing on the Illumina MiSeq platform using the Nextera XT library preparation kit (Illumina, Saffron Walden, UK). Samples were loaded and run using a 2 × 250 cycle version 2 kit. DNA samples were diluted to 0.2 ng/μl, prior to normalization and pooling. Paired-end sequencing reads were provided as FASTQ files (NU-OMICS) and subjected to downstream analysis. ORF prediction and annotations were assigned using DNA Master (Lawrence lab, Pittsburgh, PA), Glimmer (49), and GeneMark (50).

### Electron microscopy.

Purified phage were negatively stained with uranyl acetate (51) and imaged in an FEI Tecnai 12 G2 transmission electron microscope fitted with a charge-coupled-device (CCD) camera.

### Mass spectrometry.

Whole-phage samples were run into a 7-cm NuPAGE Novex 10% Bis-Tris gel (Life Technologies) at 200 V for 6 min. The total protein band was excised and digested in-gel with 0.5 μg of trypsin, overnight at 37°C. Peptides were extracted, concentrated, and loaded onto a nanoACQUITY ultraperformance liquid chromatography (UPLC) system (Waters) equipped with a nanoACQUITY symmetry C_18_, 5-μm trap (180 μm by 20 mm; Waters) and a nanoACQUITY HSS T3 1.8-μm C_18_ capillary column (75 μm by 250 mm; Waters). The nanoLC system was interfaced with a maXis HD LC-MS/MS system (Bruker Daltonics) with CaptiveSpray ionization source (Bruker Daltonics). Positive electrospray ionization-MS (ESI-MS) and MS/MS spectra were acquired using AutoMSMS mode. Instrument control, data acquisition, and processing were performed using the Compass 1.7 software (micrOTOF control, HyStar, and DataAnalysis; Bruker Daltonics). The collision energy and isolation width settings were automatically calculated using the AutoMSMS fragmentation table, with an absolute threshold of 200 counts, preferred charge states of 2 to 4, and singly charged ions excluded. A single MS/MS spectrum was acquired for each precursor, and former target ions were excluded for 0.8 min unless the precursor intensity increased 4-fold. Protein identification was performed by searching tandem mass spectra against the NCBI nr database using the Mascot search program. Matches were filtered to accept only peptides with expect scores of 0.05 or better.

### Plasmid construction.

Plasmids used in this study are listed in Table 2 and oligonucleotides in Table 3. General molecular biology techniques, including plasmid DNA preparation, genomic DNA preparation, restriction endonuclease digestion, and agarose gel electrophoresis, were performed as described by Sambrook et al. (44). In-fusion cloning technology (Clontech) was generally used for construction of plasmids. PCR-amplified DNA was generated using primers with infusion tags for insertion into plasmid vectors, which had been cut with restriction endonucleases. The ϕJoe integrating plasmid, pCMF92, was created by infusion cloning of the ϕJoe *int* gene and *attP* region, obtained by PCR with Joe Int-attP F/R primers and ϕJoe genomic DNA as the template, into the 3.1-kbp EcoRI-SphI fragment from pSET152. Plasmid pCMF91 was generated by inserting the amplified *attP* site prepared using ϕJoe genomic DNA as the template and primers Joe *attP* F/R into EcoRI-linearized pSP72. The integration sites in S. coelicolor were named *attLsc* and *attRsc* and were amplified from S. coelicolor genomic DNA (gDNA) using Joe *attB1* F/R and Joe *attB2* F/R. The *attB* site from S. venezuelae (*attBsv*) was amplified using S. venezuelae gDNA with Joe *attB Sv* F*/*Joe *attB* R primers. All three attachment sites were inserted into EcoRI-linearized pGEM7 to produce pCMF90, pCMF94, and pCMF95, respectively. The reconstituted S. coelicolor *attB* sequence (*attBsc*) was prepared from two complementary oligonucleotides, Joe *attB* Recon F and Joe *attB* Recon R (Ultramer primers; IDT) that were annealed and inserted into EcoRI-linearized pGEM7 to produce pCMF97. pCMF98 contains the ϕJoe *attLsv* and *attRsv* sites in head-to-tail orientation and was isolated by transformation of an *in vitro* recombination reaction between pCMF91 (containing ϕJoe *attP*) and pCMF95 (containing *attBsv*) into E. coli. The *attLsv* and *attRsv* sites in pCMF98 were confirmed by Sanger sequencing (GATC Biotech Ltd., London, UK). The recombination reporter plasmid pCMF116 was constructed by PCR amplification of *lacZ*α using E. coli MG1655 gDNA (52) as the template and Joe BzP forward and reverse primers containing the ϕJoe *attBsv* and ϕJoe *attP* sequences, respectively; this resulted in the *attBsv* and *attP* sites flanking the *lacZ*α gene in head-to-tail orientation. The amplified DNA was inserted into XmnI-linearized pACYC184. pCMF103 was constructed in the same way as pCMF116 except that Joe LzR F/R primers containing the ϕJoe *attLsv* and *attRsv* sites were used.

**TABLE 2:** Plasmids used in this study

Plasmid	Description	Resistance^*a*^	Reference or source
pSET152	ϕC31 *int* + *attP* integrating vector	Apra	64
pEHISTEV	Expression vector, T7 promoter, C-terminal His_6_, TEV cleavage site	Kan	65
pETFPP_2	Expression vector; His_6_-MBP-3c cleavage site	Kan	66
pBAD-HisA	Expression vector, araBAD inducible promoter	Amp	Invitrogen
pCMF87	pEHISTEV + ϕJoe *int* (gp53)	Kan	This study
pCMF90	pGEM7 + S. coelicolor *attRsc* (274 bp)	Amp	This study
pCMF91	pSP72 + ϕJoe *attP* (354 bp)	Amp	This study
pCMF92	ϕJoe int + *attP* integrating vector; pSET152	Apra	This study
pCMF94	pGEM7 + S. coelicolor *attLsc* (419 bp)	Amp	This study
pCMF95	pGEM7 + S. venezuelae *attBsv* (462 bp)	Amp	This study
pCMF96	pETFPP_2 + ϕJoe MBP-RDF (gp52)	Kan	This study
pCMF97	pGEM7 + S. coelicolor reconstituted *attBsc* (152 bp)	Amp	This study
pCMF98	ϕJoe *attLsv-attRsv*; pCMF91 integrated into pCMF95	Amp	This study
pCMF100	pEHISTEV + ϕJoe RDF	Kan	This study
pCMF103	pACYC184 + ϕJoe *attLsv-lacZα-attRsv*	Cm	This study
pCMF107	pBAD + ϕJoe *int*	Amp	This study
pCMF108	pBAD + ϕJoe RDF + *int* coexpression	Amp	This study
pCMF116	pACYC184 + ϕJoe *attBsv-lacZ*α-*attP*	Cm	This study
pCMF117	pEHISTEV + ϕJoe RDF + *int* coexpression	Kan	This study
pGEM7	General cloning vector	Amp	Promega
pSP72	General cloning vector; accession no. X65332	Amp	Promega
pACYC184	General cloning vector; accession no. X06403	Cm	67
pUZ8002	Conjugation helper plasmid; RK2 derivative with defective *oriT*	Kan	68

a Apra, apramycin; Kan, kanamycin; Amp, ampicillin; Cm, chloramphenicol.

**TABLE 3:** Primers used in this study

Primer	Sequence (5′ to 3′)
Joe Int-*attP* F	CCGTCGACCTGCAGGCATGCCGTTCCCGCAGGTCAGAGC
Joe Int-*attP* R	ACATGATTACGAATTCTGTGGATCAGAACGTCTCGG
Joe H6-Int F	TTTCAGGGCGCCATGATGAGTAACCGACTACATG
Joe H6-Int R	CCGATATCAGCCATGTCAGAACGTCTCGGCGAAG
Joe *attP* F	TACCGAGCTCGAATTAAGACCGTCTCAGCCAGG
Joe *attP* R	TATCATCGATGAATTTCAGTGAAGACGGACAGG
Joe *attB1* F	CCGGGGTACCGAATTTGTGACGTCAGCCACAGC
Joe *attB1* R	TAGACTCGAGGAATTGACAAGGAGTGGCTCTGG
Joe *attB2* F	CCGGGGTACCGAATTGACTGCGTGCCGTCAGCC
Joe *attB2* R	TAGACTCGAGGAATTCGTCGTGTCGTCTGTCAG
Joe *attB* Sv F	CCGGGGTACCGAATTACCAGGTGGTGGATGAGC
Joe *attB* Recon F	TAGACTCGAGGAATTACCTTGATCTCGGTGTCCATCGCCGGGCAGACGCCGCAGTCGAAGCACGG
Joe *attB* Recon R	CCGGGGTACCGAATTGACAAGGAGTGGCTCTGG
Joe MBP-gp52 F	TCCAGGGACCAGCAATGAACGGACAGATCCTGG
Joe MBP-gp52 R	TGAGGAGAAGGCGCGCTACACCCAGCGCACCGA
CleF	CGCGCCTTCTCCTCACATATGGCTAGC
CleR	TTGCTGGTCCCTGGAACAGAACTTCC
Joe H6-gp52 F	TTTCAGGGCGCCATGAACGGACAGATCCTGGAG
Joe H6-gp52 R	CCGATATCAGCCATGCTACACCCAGCGCACCGA
Joe pBAD Int F	GAGGAATTAACCATGAGTAACCGACTACATG
Joe pBAD Int R	TGAGAACCCCCCATGTCAGAACGTCTCGGCGAAG
Joe pBAD gp52 F	GAGGAATTAACCATGAACGGACAGATCCTGGAG
Joe pBAD Int Co-Ex F	AGTGGTAGGTTCCTCGCCATG
Joe pBAD gp52 R	GAGGAACCTACCACTCTACACCCAGCGCACCGA
Joe LzR F	GGGTGTCAGTGAAGTAGTTGTGGCCATGTGTCCATCTGGGGGCAGACGCCGCAGTCGAAGCACGGCGATTTCGGCCTATTGGT
Joe LzR R	CCTGCCACATGAAGCGGATGTGACCCCGTCTCCATCTGCCCGCAGATGGACACCCACATCCAGATAATACGCAAACCGCCTCT
Joe BzP F	GGGTGTCAGTGAAGTATCTGGATGTGGGTGTCCATCTGCGGGCAGACGCCGCAGTCGAAGCACGGCGATTTCGGCCTATTGGT
Joe BzP R	CCTGCCACATGAAGCGGATGTGACCCCGTCTCCATCTGCCCCCAGATGGACACATGGCCACAACTAATACGCAAACCGCCTCT
SPBc H6-sprA F	CCGATATCAGCCATGGAGTTAAAAAACATTGTT
SPBc H6-sprA R	TTTCAGGGCGCCATGCTTACTACTTTTCTTAGTGG
SPBc MBP-sprB F	TCCAGGGACCAGCAATGGAACCTTACCAACGT
SPBc MBP-sprB R	TGAGGAGAAGGCGCGAAGCTTACTCTGCCTTCC
SPBc LZR F	GGGTGTCAGTGAAGTAGTGCAGCATGTCATTAATATCAGTACAGATAAAGCTGTATATTAAGATACTTACTACATATCTACGATTTCGGCCTATTGGT
SPBc LZR R	CCTGCCACATGAAGCTGGCACCCATTGTGTTCACAGGAGATACAGCTTTATCTGTTTTTTAAGATACTTACTACTTTTCTAATACGCAAACCGCCTCT

The integrase expression plasmid for protein purification, pCMF87, was constructed by insertion of a PCR fragment containing the ϕJoe *int* gene, amplified from ϕJoe gDNA using primers Joe H6-Int F/R, into NcoI-linearized pEHISTEV expression vector. ϕJoe *g52*, encoding the RDF, was PCR amplified from ϕJoe gDNA using primers Joe MBP-g52 F/R and inserted into pETFPP_2 MBP-tag expression vector linearized by PCR with CleF/R to create pCMF96. For *in vivo* recombination assays, the integrase expression plasmid pCMF107 was constructed by insertion of a PCR fragment containing the ϕJoe *int* gene and amplified from ϕJoe gDNA using primers Joe pBAD Int F/R into NcoI-linearized pBAD-HisA expression vector. A ϕJoe gp52 and integrase coexpression plasmid, pCMF108, was created by amplification of each gene using Joe pBAD gp52 F/R and Joe pBAD Int Co-Ex F/Joe pBAD Int R primers, respectively, and insertion of both PCR products simultaneously into pBAD-HisA. The coexpression insert from pCMF108 was subsequently PCR amplified using Joe H6-gp52 F/Joe H6-Int R primers and transferred to NcoI-linearized pEHISTEV to produce an alternative expression vector, pCMF117.

### Conjugation and integration of plasmids in Streptomyces.

Transfer of plasmids into Streptomyces strains was performed according to the procedures described by Kieser et al. (45). Conjugation donors were produced by introduction of plasmids into the nonmethylating E. coli strain ET12567, containing an RP4 derivative plasmid (pUZ8002), by transformation. Recipient Streptomyces spores were used at a concentration of 10^8^/ml, mixed with the E. coli donors, plated onto mannitol soya agar supplemented with 10 mM MgCl_2_ with no antibiotic selection, and incubated at 30°C overnight. Plates containing the donor cells were overlaid with 1 ml of water containing 0.5 mg of nalidixic acid (for E. coli counterselection) and antibiotic for selection of exconjugants (apramycin) before further incubation of all plates at 30°C for 3 days. Integration efficiency was calculated as the number of apramycin-resistant colonies/10^8^ CFU (8).

### Protein purification.

E. coli BL21(DE3) containing the relevant expression plasmid was grown (37°C with agitation) in 500 ml of 2YT medium (1.6%, [wt/vol] tryptone, 1.0%, [wt/vol] yeast extract, 0.5 [wt/vol] NaCl) to mid-exponential-growth phase. The cultures were rapidly chilled on ice for 15 min, IPTG was added (final concentration, 0.15 mM), and the cultures were further incubated (17°C for 16 h, with agitation). Cells were harvested by centrifugation, resuspended in 20 ml of lysis buffer (1 M NaCl, 75 mM Tris [pH 7.75], 0.2 mg/ml lysozyme, 500 U of BaseMuncher endonuclease; Expedeon Ltd.), and incubated on ice (30 min). The cells were lysed by sonication, and debris was removed by centrifugation (18,000 × *g*, 5 min, 4°C). The supernatant was applied to a 5-ml HisTrap FF crude column that had been preequilibrated with binding buffer (20 mM sodium phosphate, 0.5 M NaCl, 20 mM imidazole [pH 7.4]) on an Äkta pure 25 chromatography system (GE Healthcare). Bound His-tagged protein was eluted with a step gradient of binding buffer containing 125 mM and 250 mM imidazole. Imidazole was removed from the eluted fractions by pooling the fractions containing the desired protein and applying the pooled solutions to a HiPrep 26/10 desalting column (GE Healthcare) equilibrated with imidazole-free binding buffer. Finally, the protein extracts were subjected to size exclusion chromatography on a HiLoad 16/60 Superdex column. Purified protein fractions were concentrated in a Vivaspin sample concentrator (GE Healthcare) and quantified by absorbance at 280 nm on a NanoDrop spectrophotometer (Thermo Scientific). Protein analysis was performed by denaturing acrylamide gel electrophoresis using premade gels (4 to 12% gradient acrylamide; Expedeon Ltd.); gels were stained with InstantBlue (Expedeon, Ltd.). For storage, an equal volume of 100% glycerol was added to protein samples before freezing at −80°C.

### *In vitro* assays.

Recombination reactions (final volume, 20 μl) were carried out in ϕC31 RxE buffer (10 mM Tris [pH 7.5], 100 mM NaCl, 5 mM dithiothreitol [DTT], 5 mM spermidine, 4.5% glycerol, 0.5 mg/ml bovine serum albumin [BSA]) (53), Bxb1 RxE buffer (20 mM Tris [pH 7.5], 25 mM NaCl, 1 mM DTT, 10 mM spermidine, 10 mM EDTA) (23), or TG1 RxE (as Bxb1 RxE plus 0.1 mg/ml BSA) (54). Integrase and RDF proteins were added at the concentrations indicated for each experiment. Plasmids containing the recombination substrates were used at 100 ng per reaction. Reactions were either incubated at 30°C for 2 h (to reach steady state) or for specified times. Reactions were stopped by heat (10 min, 75°C), the buffer was adjusted to be compatible with restriction enzymes, and the plasmids were digested with XhoI (NEB). The linearized reaction mixtures were run on a 0.8% agarose gel, and the relative band intensities were measured to assess activity. Recombination efficiencies were calculated as the intensity of product band(s)/sum intensity of all bands.

### Bioinformatics.

The ϕJoe genome was visualized using DNAPlotter (55). The *attB* DNA alignment and logo consensus sequence were created with Jalview (56). Protein sequence alignments for visual presentation were produced using the Clustal W (57) program within the BioEdit suite (58). Protein alignments for phylogenetic analysis were produced using Clustal Omega (59), and maximum likelihood trees were created in MEGA6 (60). The BLOSUM62 similarity matrix was used for protein alignment and annotation (61). Structural alignment of the small RDF proteins was carried out with Promals3D (62). Band densities for *in vitro* assays were measured using the Fiji GelAnalyzer module (63).

### Accession number(s).

The annotated genome sequence of ϕJoe was submitted to GenBank (accession number: KX815338). Accession numbers for all sequences used here are also provided in Table S2.

## Supplementary Material

Supplemental material
