# Supplementary material for: Genome Integration and Excision by a New Streptomyces Bacteriophage, ϕJoe
Source: Appl Environ Microbiol. 2017 Feb 15;83(5):e02767-16. doi: 10.1128/AEM.02767-16 (PMC5311408; doi:10.1128/AEM.02767-16)
Supplement: Supplemental material [file supp_83_5_e02767-16__index.html]

Supplemental material 

# Genome Integration and Excision by a New Streptomyces Bacteriophage, ϕJoe

## Supplemental material

- Supplemental file 1 -

  Schematic of ϕJoe integrating plasmid pCMF92 (Fig. S1), Circos comparison (Fig. S2), maps of the substrate and product plasmids (Fig. S3), representative agarose gels (Fig. S4), molecular phylogenetic analyses (Fig. S5), alignment of putative RDFs carried by representative SCO2603-like integrase encoding MGEs (Fig. S6), prediction of coiled coil motifs and Swiss model prediction (Fig. S7), ϕJoe structural proteome (Table S1), and accession numbers/protein IDs (Table S2).

  PDF, 1004K
