# Supplementary material for: Genome Integration and Excision by a New Streptomyces Bacteriophage, ϕJoe
Source: Appl Environ Microbiol. 2017 Feb 15;83(5):e02767-16. doi: 10.1128/AEM.02767-16 (PMC5311408; doi:10.1128/AEM.02767-16)
Supplement: Supplemental material [file AEM.02767-16_zam999117694s1.pdf]

Supplemental Figures

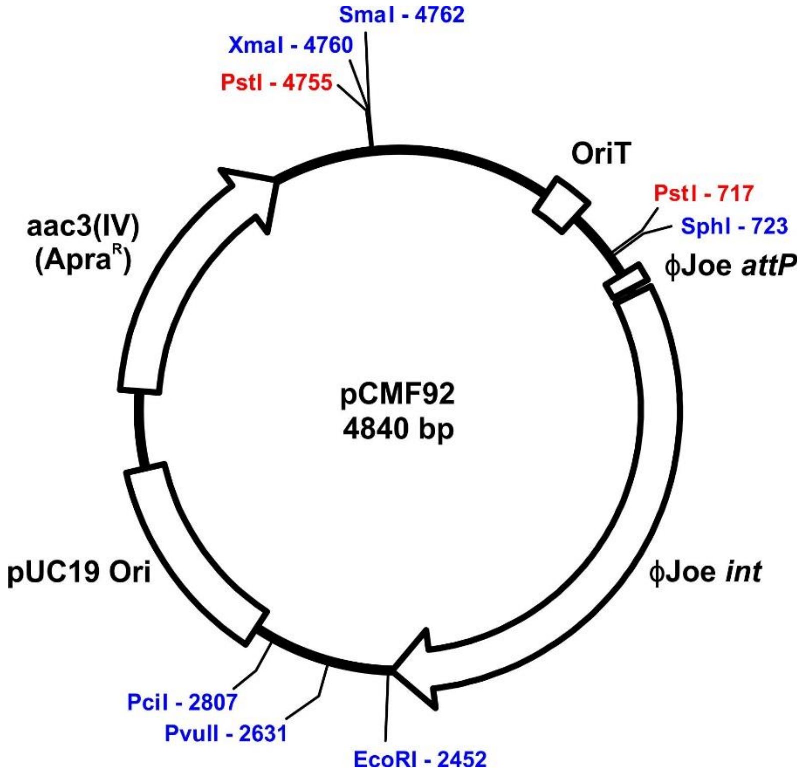

Figure S1: Schematic of the ϕJoe integrating plasmid, pCMF92. Locations of the ϕJoe *int* gene and *attP* site are indicated along with relevant plasmid features – origin of transfer (**OriT**), apramycin resistance gene (**aac3(IV)**) and *E. coli* replication origin (**pUC19 Ori**). After integration of the plasmid, the PstI sites shown were used to confirm that integration into the *S. coelicolor* genome had occurred and to identify the *attB* sites by recircularization and recovery of the intervening DNA. Unique restriction sites in intergenic regions of the plasmid are shown in blue.

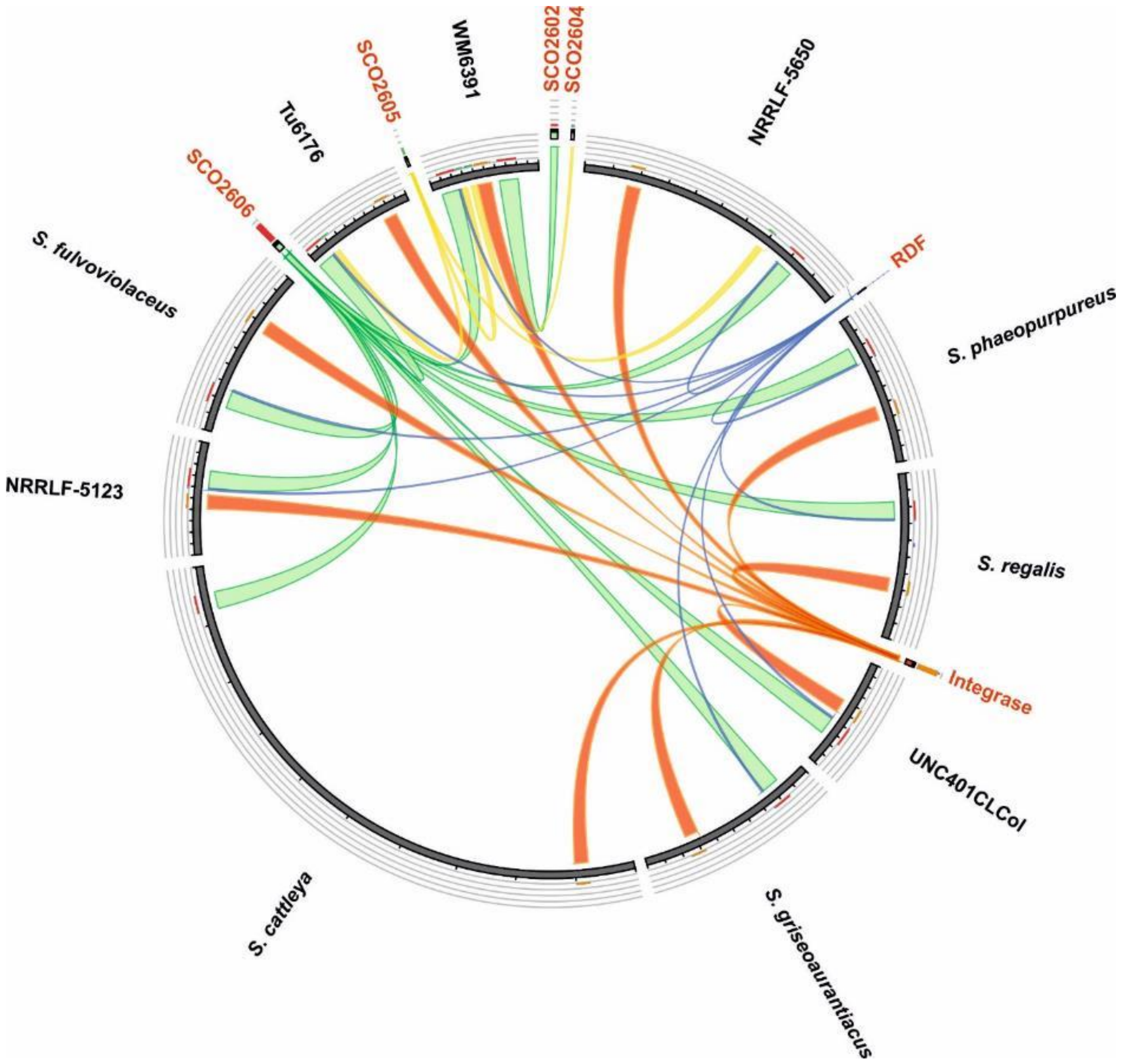

Figure S2: Circos comparison of the *S. coelicolor* SCO2603-encoding putative mobile genetic element (MGE) to related MGEs in other strains. A tBlastn alignment was carried out using six protein queries from *S. coelicolor* - SCO2603 (**Integrase**), RDF (previously unassigned, located between SCO2605 and SCO2606), SCO2604/SCO2605 (hypothetical proteins within the putative MGE) and SCO2602/SCO2606 (genes flanking the putative MGE). Ten nucleotide subject sequences from different species (as labelled) were chosen to represent the broad diversity of sequence content detected. The E-value cut-off was set to $1x10^{-5}$ and the HSPs to 100. Ribbons are coloured by query protein; integrase (orange), RDF (blue), flanking genes (green), hypothetical genes within the putative MGE (Yellow). The histograms above each genome are coloured to reflect relative homology to the ϕJoe sequence based on Blast score (Red>Orange>Green>Blue).

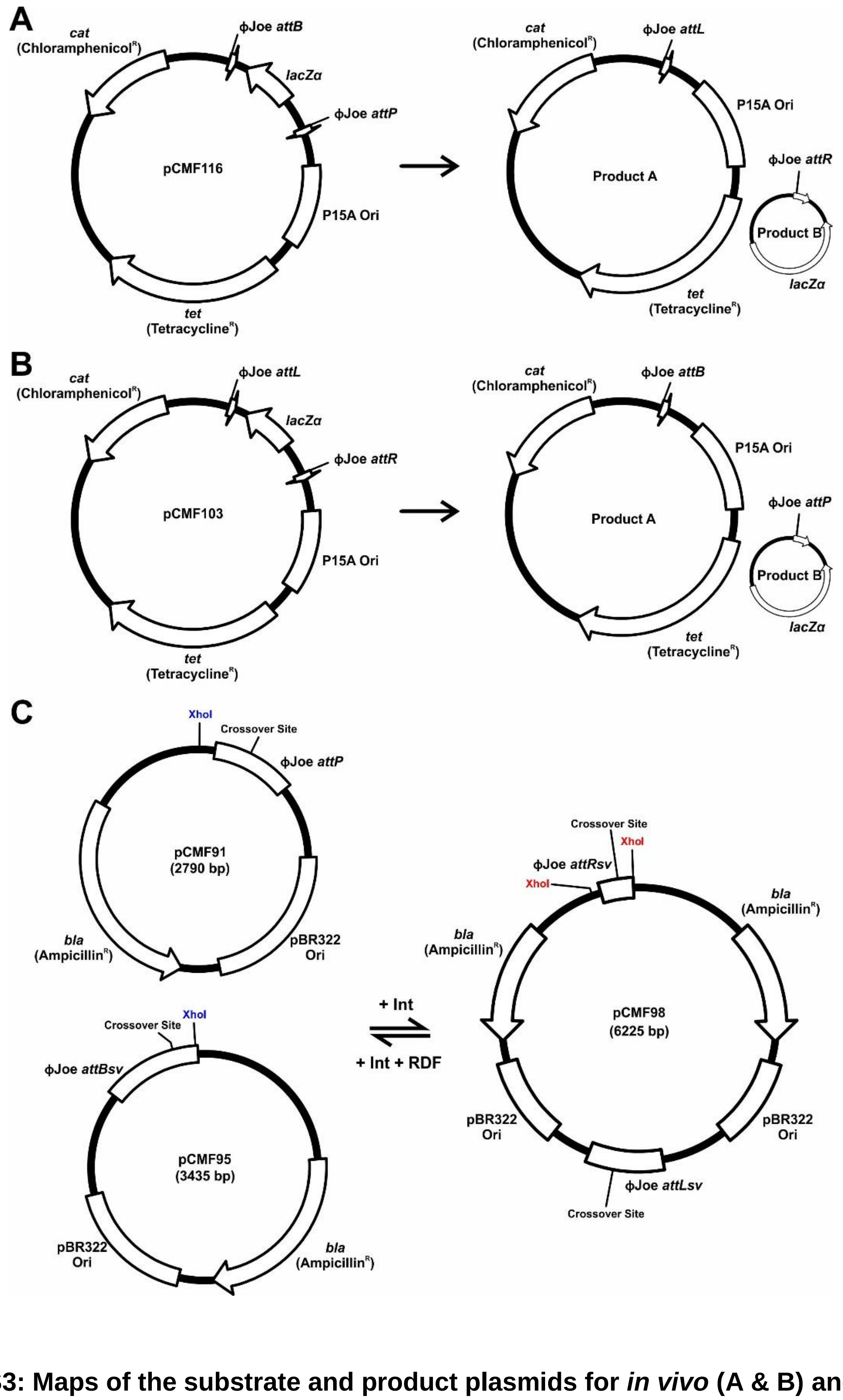

Figure S3: Maps of the substrate and product plasmids for *in vivo* (A & B) and *in vitro* (C) recombination reactions. *In vivo* recombination of *attB* and *attP* (**A**) or *attL* and *attR* (**B**) by the ϕJoe integrase excises the intervening *lacZα* gene to produce a replicating plasmid (**Product A**) and a non-replicating *lacZα* circular DNA (**Product B**), the latter of which is subsequently lost. **C.** *In vitro* recombination of *attB* and *attP* containing plasmids produces a co-integrant plasmid (**pCMF98**). The reaction can be reversed to reform the substrates in the presence of the RDF. The substrate and product plasmids can be distinguished and quantified on an agarose gel after XhoI cleavage at the indicated sites.

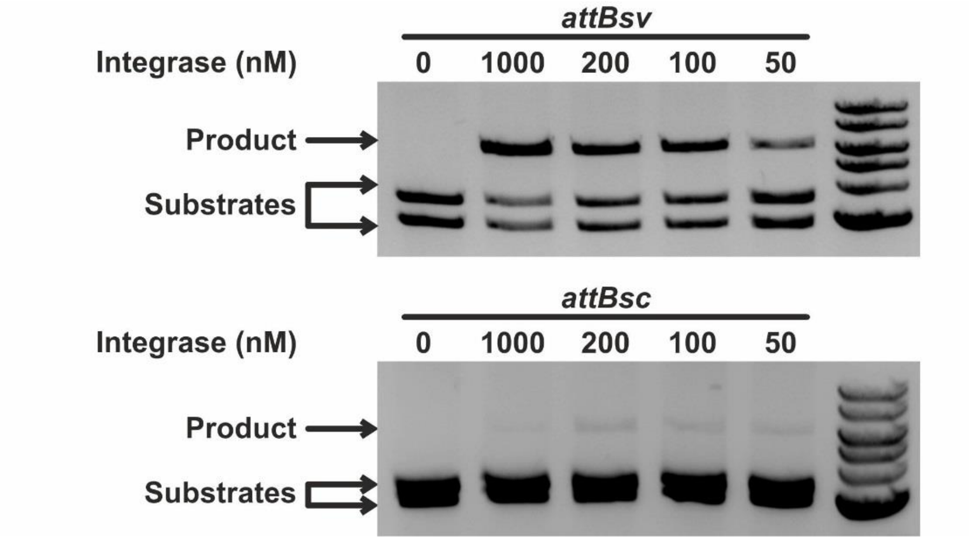

Figure S4: Representative agarose gel showing ϕJoe integrase *in vitro* integration reactions with *S. venezuelae attB* (***attBsv***) or the reconstituted *S. coelicolor attB* (***attBsc***) as substrates. The concentration of ϕJoe Integrase for each reaction is indicated above the image. Reactions were stopped after 2 h. A very faint band was present for the recombined *attBsc* x *attP* plasmid, with a peak equivalent to ~1.5% of total DNA when 200 nM integrase was used, compared to substantial recombination for *attBsv* at all Int concentrations.

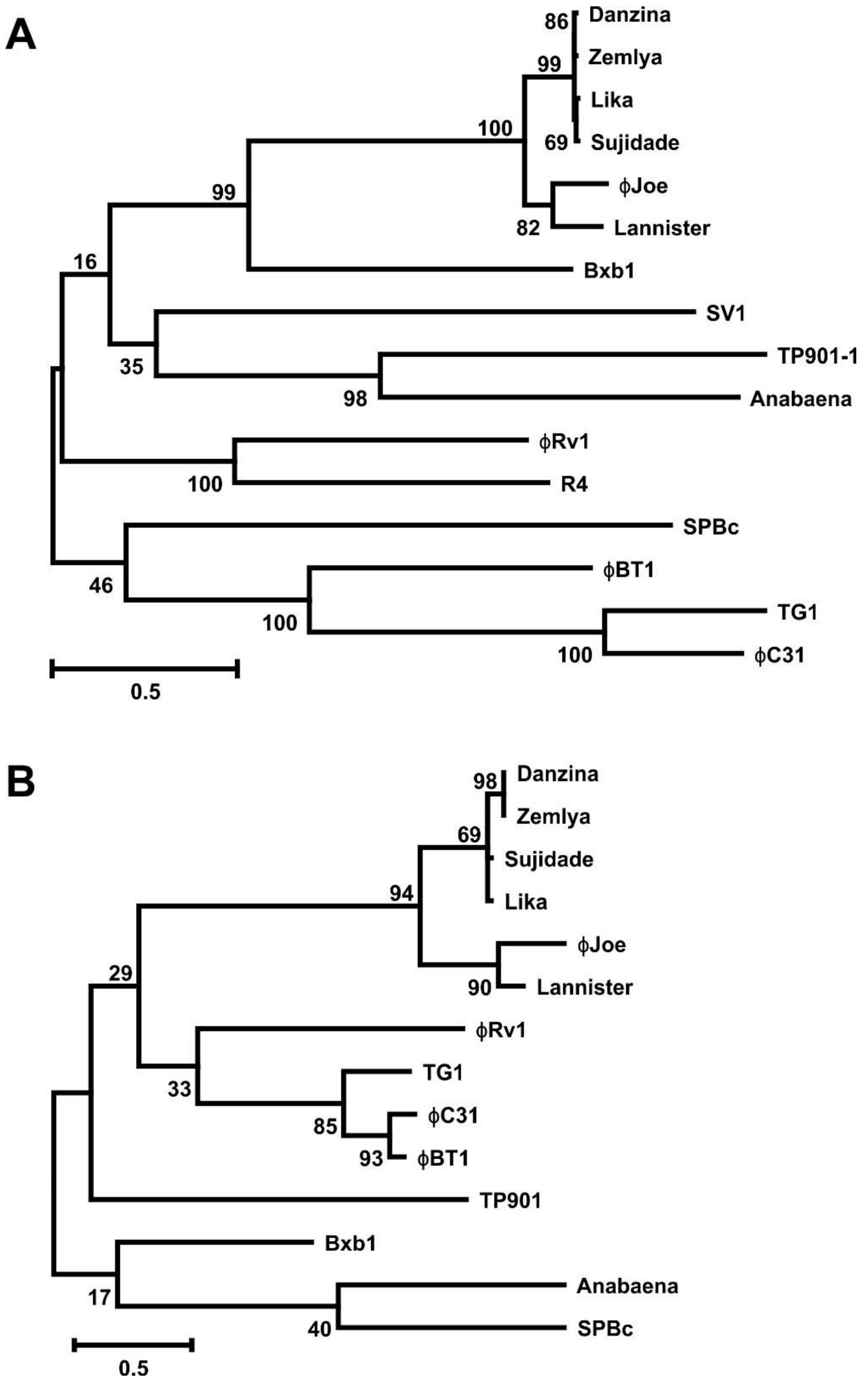

Figure S5: Molecular phylogenetic analyses of (A) serine integrases and (B) RDFs. Protein sequences were aligned using Clustal Omega and evolutionary analyses were conducted in MEGA6 (2). The evolutionary history was inferred using the Maximum Likelihood method (3). Trees with the highest log likelihood (**A:** -16440.7661 & **B:** -3155.4478) are shown. The percentage of trees in which the associated taxa clustered together is shown next to the branches. Initial tree(s) for the heuristic search were obtained automatically by applying Neighbor-Join and BioNJ algorithms to a matrix of pairwise distances estimated using a JTT model, and then selecting the topology with superior log likelihood value. The tree is drawn to scale, with branch lengths measured in the number of substitutions per site. The analysis involved 16 and 14 amino acid sequences, respectively. A total of 782 positions for the integrases and 300 for the RDFs were in the final dataset after all positions containing gaps and missing data were eliminated.

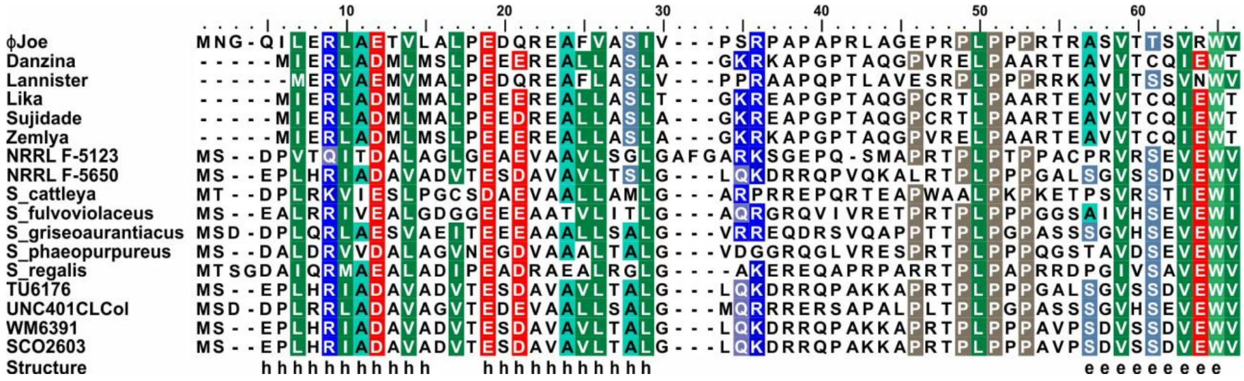

Figure S6: Alignment of putative RDFs carried by representative SCO2603-like integrase encoding MGEs. The sequences shown are not an exhaustive survey of the RDFs from MGEs that encode a SCO2603-like integrase but are intended to represent a diverse selection of the MGEs, in terms of size and gene content (see Fig. S3). Shading is based on the BLOSUM62 similarity matrix with a 70% threshold assigned. ϕJoe, Danzina, Lannister, Lika, Sujidade and Zemlya RDF sequences are included for comparison and are discussed in the main text. All other sequences were identified in published *Streptomyces* genomes. Where only a strain designation is given (e.g. WM6319) it is because the species was not stipulated in the genome database. The original sequence identified in *S. coelicolor* A3(2) is labelled SCO2603. Other strain designations are as follows: *S. cattleya* 46488, *S. fulvoviolaceus* NRRL B-2870, *S. griseoaurantiacus* M045, *S. phaeopurpureus* DSM 40125 and *S. regalis* NRRL3151. Structure prediction is shown beneath the alignment (**Structure**) where h = alpha helix and e = beta sheet.

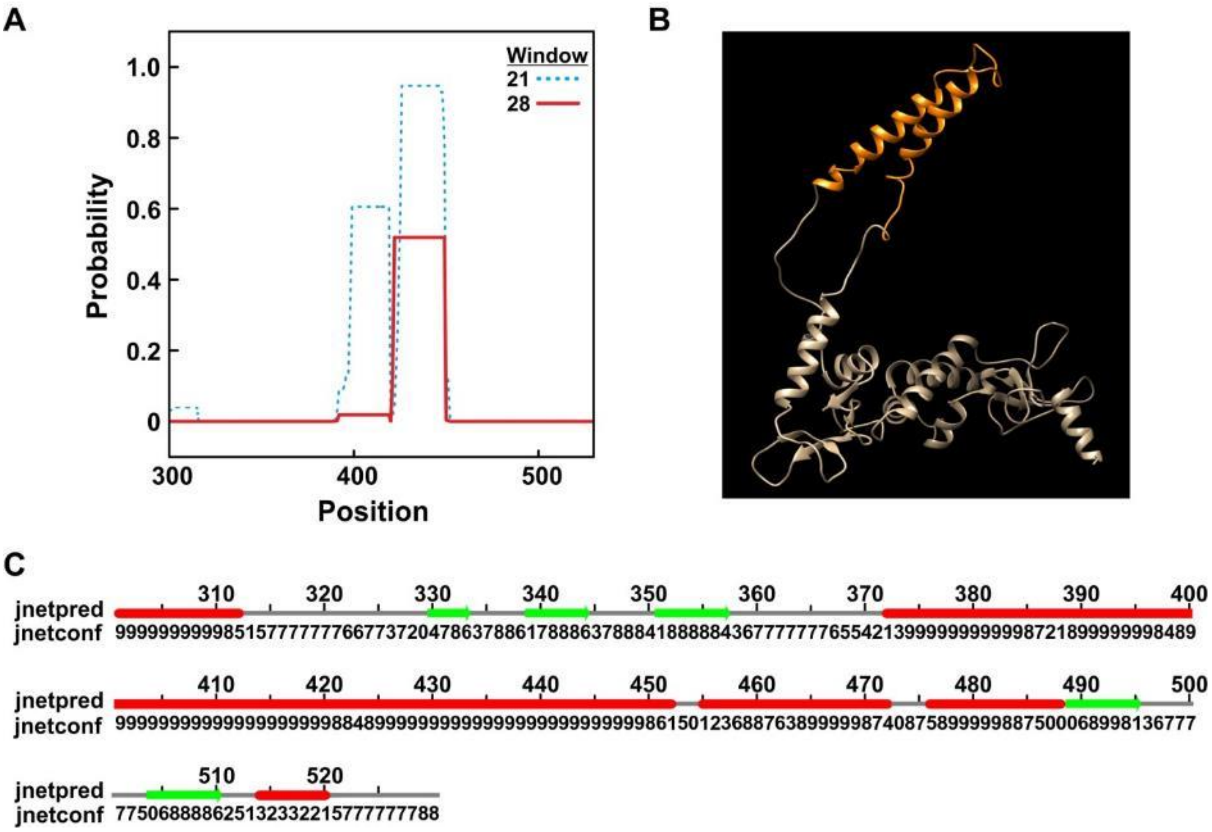

Figure S7: A. Prediction of coiled coil motifs in ϕJoe Int C-terminal domain using COILS (4). The amino acid position is listed on the X-axis and the probability score on the Y-axis. Predictions were made using window sizes of 21 or 28 amino acids. **B.** Swiss model prediction of the ϕJoe Int C-terminal domain structure using A118 Int as a template (PDB: 4kis) (5). The putative coiled coil domain is highlighted in orange and corresponds to residues A395-T453. The Global Model Quality Estimation for the model is 0.29 and the sequence identity is 16.55%. **C.** JPred4 secondary structure prediction for ϕJoe Int C-terminal domain (6). Coils are shown as red tubes and sheets as green arrows, the amino acid positions are indicated above the plots and confidence values below (scale 0-9).

Supplementary Table S1. ϕJoe structural proteome determined by MS:MS

Gene	Annotation	Mass	Score	Matches	Sequences	emPAI^
**g09**	Portal	52019	1401	33	24	3.23
**g10**	Unknown Function	40058	164	4	4	0.35
**g11**	Scaffold Protein	19449	17	1	1	0.16
**g12**	Major Capsid	38928	3689	107	55	98.96
**g13**	Unknown Function	16663	468	8	8	3.06
**g14**	Head-Tail Adaptor	12773	126	2	2	0.57
**g16**	Unknown Function	18449	337	9	8	2.57
**g17**	Unknown Function	25499	165	3	2	0.26
**g20**	Tail Tape Measure	153936	324	9	8	0.17
**g21**	Unknown Function	30343	153	3	3	0.34
**g22**	Unknown Function	43342	428	6	6	0.51
**g24**	Unknown Function	40324	441	7	6	0.56
**g27**	Unknown Function	44698	326	6	5	0.40
**g28**	Unknown Function	12903	17	1	1	0.25

^ The Exponentially Modified Protein Abundance Index (emPAI) offers approximate, label-free, relative quantitation of the proteins in a mixture based on protein coverage by the peptide matches in a database search result (1)

Supplementary Table S2. Accession numbers/protein IDs for sequences used in this study

Species	Genome	Integrase	RDF
**Bacteriophage**			
**ϕJoe**	KX815338	APC43293	APC43292
**ϕCAM**	JX889246	AFV51369	u/k
**Lannister**	NC_028827	YP_009200991	YP_009200990
**Zemlya**	NC_021339	YP_008060284	n/a
**Lika**	NC_021298	YP_008050906	n/a
**Sujidade**	NC_021304	YP_008051452	n/a
**Amela**	NC_028904	YP_009208329	u/k
**Verse**	KT186229	AKY03881	u/k
**Danzina**	KT124228	AKY03507	AKY03506
**R4**	NC_019414	YP_006990167	u/k
**ϕRv1^**	NC_000962	NP_216102	NP_216100
**Bxb1**	NC_002656	NP_075302	NP_075314
**SV1**	NC_018848	YP_006906969	u/k
**TP901-1**	NC_002747	NP_112664	NP_112670
**SPBc^**	NC_000964	NP_390049	NP_389863
**ϕBT1**	NC_004664	NP_813744	NP_813719
**ϕC31**	NC_001978	NP_047974	NP_047948
**TG1**	NC_018853	YP_006907228	YP_006907201
***Anabaena variabilis* ATCC 29413^**	CP000117	ABA25082	ABA23430
**Putative Mobile Genetic Elements**			
**Streptomyces coelicolor A3(2)**	NC_003888	NP_626840	n/a
***S. phaeopurpureus* DSM 40125**	KQ948183	KUM72918	KUM72731
***Streptomyces* WM6391**	JXWX01000030	KKD13794	KKD13791
***Streptomyces* UNC401CLCol**	NZ_JMLN01000030	WP_028961125	WP_028961127
***Streptomyces* Tu 6176**	NZ_KK106990	WP_017944909	WP_037893069
***Streptomyces regalis* NRRL 3151**	NZ_LLZG01000265	WP_062705520	WP_062705458
***S. fulvoviolaceus* NRRL B-2870**	NZ_JOEY01000013	WP_052424710	WP_030601555
***S. griseoaurantiacus* M045**	NZ_AEYX01000002	WP_040893440	WP_040893426
***S. cattleya* DSM 46488**	NC_017586	WP_041825041	WP_014142485
***Streptomyces* NRRL F-5650**	NZ_JOGV01000009	WP_031038341	WP_031038376
***Streptomyces* NRRL F-5123**	NZ_JOHY01000007	WP_052397223	WP_031514555

^ = Prophage/MGE located within a bacterial genome sequence
u/k = unknown; RDF not known at this time
n/a = not applicable; predicted RDF gene is not annotated in the database

References

1. **Ishihama Y**, **Oda Y**, **Tabata T**, **Sato T**, **Nagasu T**, **Rappsilber J**, **Mann M**. 2005. Exponentially Modified Protein Abundance Index (emPAI) for Estimation of Absolute Protein Amount in Proteomics by the Number of Sequenced Peptides per Protein. Mol Cell Proteomics **4**:1265–1272.
2. **Tamura K**, **Stecher G**, **Peterson D**, **Filipski A**, **Kumar S**. 2013. MEGA6: Molecular evolutionary genetics analysis version 6.0. Mol Biol Evol **30**:2725–2729.
3. **Jones DT**, **Taylor WR**, **Thornton JM**. 1992. The rapid generation of mutation data matrices from protein sequences. Bioinformatics **8**:275–282.
4. **Lupas a**, **Van Dyke M**, **Stock J**. 1991. Predicting coiled coils from protein sequences. Science **252**:1162–4.
5. **Biasini M**, **Bienert S**, **Waterhouse A**, **Arnold K**, **Studer G**, **Schmidt T**, **Kiefer F**, **Cassarino TG**, **Bertoni M**, **Bordoli L**, **Schwede T**. 2014. SWISS-MODEL: Modelling protein tertiary and quaternary structure using evolutionary information. Nucleic Acids Res **42**:W252-8.
6. **Drozdetskiy A**, **Cole C**, **Procter J**, **Barton GJ**. 2015. JPred4: A protein secondary structure prediction server. Nucleic Acids Res **43**:W389–W394.
